# Analysis of compliance issues and influencing factors in the management of BSL-2 laboratories for pathogenic microorganisms in Lishui, China

**DOI:** 10.3389/fbioe.2025.1637056

**Published:** 2025-08-08

**Authors:** Ying Cong, Jinkai Li, Dingshuo Lou, Jianliang Zhu, Deyong Zhang, Dongqing Cheng, Xiuying Chen

**Affiliations:** ^1^ Lishui Center for Disease Control and Prevention, Lishui, China; ^2^ School of Medical Technology and Information Engineering, Zhejiang Chinese Medical University, Hangzhou, China

**Keywords:** biosafety, pathogenic microorganisms, quality control, online supervision systems, laboratory management

## Abstract

**Background:**

The management of biosafety laboratories for pathogenic microorganisms is directly related to public health and the effectiveness of biological experiments. However, persistent non-compliance issues in biosafety level 2 (BSL-2) laboratory management remain a challenge. This study aimed to assess the quality control of BSL-2 laboratories for pathogenic microorganisms in Lishui.

**Methods:**

By combining the Biosafety Online Supervision Systems and on-site inspections, this study assessed the quality control of 73 medical institutions and the 128 biosafety laboratories under their management in Lishui.

**Results:**

The results discovered that the 73 medical institutions had low compliance rates in several fields: Responsibilities of the biosafety management (80.82%), development of system documentation (65.75%), risk assessment (84.56%), training of laboratory personnel (82.19%), and biosafety labeling (52.05%). Additionally, 128 laboratories had low pass rates for the access control management system (85.94%), hand/eye wash and shower stations (85.94%), and biosafety cabinet operations (89.06%).

**Conclusion:**

This study demonstrates that future efforts should focus on strengthening laboratory personnel training and implementing biosafety management responsibilities to ensure safe and regulation-compliant operations in laboratories handling pathogenic microorganisms.

## 1 Introduction

Laboratory biosafety management, a critical component of the public health system, is defined as the aggregation of measures designed to prevent the unintentional exposure of biohazardous materials (World Health Organization, 2020), and it plays a pivotal role in the disease surveillance system (Brown et al., 2015; Edwards and Jeggo, 2010). The significance of laboratory biosafety management has been accentuated in the context of the increasing frequency of public health events. Furthermore, it could ensure the protection of laboratory personnel from potentially harmful biological agents (Global Health Security Agenda, 2014). Nevertheless, despite the existence of numerous pertinent standards and guidelines for biosafety management in laboratories, insufficient comprehension among personnel has directly elevated risks of biosafety incidents (Pašalić et al., 2022). As highlighted in a study, 80% of laboratory infections are attributable to human factors, underscoring the significance of rigorous adherence to standards and guidelines for laboratory biosafety management (Mohsen and Dpagh, 2015).

Moreover, disease outbreaks resulting from laboratory accidents can exert a considerable impact on economic development (Siengsanan-Lamont and Blacksell, 2018) and substantially impede progress toward the United Nations Sustainable Development Goals (Min and Perucci, 2020). It has been demonstrated that a substantial proportion of laboratories faced operational and sustainability challenges prior to the COVID-19 pandemic (Davies et al., 2017), which have subsequently emerged as critical risks to global public health systems (Hamilton et al., 2018). It is noteworthy that a significant proportion of high-protection laboratories are situated in or planned for urban centers, where the accidental or deliberate release of pathogens can lead to serious consequences (Klotz and Sylvester, 2014). Countries that have established or are planning to establish BSL-3 and BSL-4 laboratories have received low ratings on the indicators used to assess the biosafety of their laboratories (Lentzos and Koblentz, 2023). In addition, the proliferation of BSL-3 and BSL-4 facilities in recent years has led to an increase in the risk of biosafety incidents, underscoring the necessity for timely and pertinent training for laboratory personnel (Morrison and Simoneau, 2023). This training is crucial for enhancing biosafety awareness and enabling the effective fulfillment of their supervisory roles in the development and implementation of pathogen research (Qasmi et al., 2022).

To address these concerns, the Laboratory Biosafety Manual, published by the World Health Organization (WHO) in 2020, advocates laboratory biosafety management based on current knowledge to mitigate the risk of pathogen escape (World Health Organization, 2020), particularly in resource-limited laboratory settings (Blacksell et al., 2023). The global “risk-driven” strategy similarly demonstrates the significance of building on the manual to enhance laboratory biosafety management (Kojima et al., 2018). Recent studies have underscored the biosafety imperative in animal experimentation, which stems from persistent occupational exposure risks in which researchers routinely handle pathogens or have direct contact with infected specimens during experimental procedures (Alderman et al., 2018). The previous COVID-19 pandemic further demonstrated the magnitude of laboratory biosafety management in preventing the further transmission of emerging pathogens (Lippi and Plebani, 2020).

In China, the development of legal and regulatory systems for laboratory biosafety management was formally initiated in the 1980s (Cao, 2021). Additionally, the management of biosafety in laboratories has risen to the level of a national strategy, in response to the increasing number of public health emergencies (Chen et al., 2020). Nonetheless, laboratories in healthcare delivery organizations (HDOs) and public health authorities (PHAs) face persistent biosafety management challenges. In addressing these challenges, Zhejiang Province has pioneered the establishment of the “Provincial Laboratory Biosafety Online Supervision Systems.” This system allows the supervisory authority to monitor laboratory biosafety in real time, identify and address potential risks in a timely manner, and effectively prevent and control biosafety incidents. In addition, the system can detect potential biosafety risks and trends by analyzing big data, providing a scientific basis for decision-making, thereby significantly improving supervision and data accuracy (Gao et al., 2024). Furthermore, it is worth noting that for the common issues identified in biosafety management, we should refer to the Zhejiang Provincial Local Standard Specifications for the Evaluation of Biosafety Laboratory Management (DB33/T 2540-2022) to achieve the standardization of laboratory construction, management, operation, transportation, decontamination, and waste disposal.

This study aimed to analyze the management level of BSL-2 laboratories for pathogenic microorganisms in Lishui from 2023 to 2024, using the Provincial Laboratory Biosafety Online Supervision System, in order to provide a basis for improving biosafety management and serve as a reference for other regions and laboratories.

## 2 Materials and methods

### 2.1 Data collection

In this study, data were collected on compliant and non-compliant items for 26 assessment indicators from 73 healthcare institutions and 19 indicators from 128 affiliated laboratories by using the Zhejiang Provincial Laboratory Biosafety Online Supervision Systems. These inspections, covering 9 counties (districts) and cities in Lishui, China from 2023 to 2024, were conducted through random sampling by provincial and municipal quality control centers. The healthcare institutions were categorized into three groups: HDOs (general hospitals, private hospitals, and community health centers), PHAs (maternal and child health hospitals and CDCs), and others. The laboratories were classified into the following types: microbiology, biochemistry, immunology, blood transfusion, pathology, and outpatient departments.

### 2.2 Laboratory self-inspections and external quality control assessments

An assessment framework comprising 45 criteria across eight domains was established through the Zhejiang Provincial Pathogenic Microorganism Laboratory Biosafety Assessment System (https://syspj.wsjkw.zj.gov.cn:888) to achieve closed-loop management of biosafety laboratories. This digital platform integrated self-inspections, quality control assessments, and post-assessment corrections. All designated laboratories were required to complete self-assessments and corrective actions within specified timelines, ensuring 100% institutional coverage. Based on submitted self-reports, on-site assessments were conducted through stratified random sampling by provincial and municipal quality control centers. Assessment teams consisted of 3–5 experts specializing in laboratory biosafety management and clinical microbiology, recruited from hospitals and CDCs.

### 2.3 Content of quality control assessments

The quality control assessments during 2023–2024 were conducted according to the Zhejiang Provincial Standard Biosafety Laboratory Management Assessment Specifications (DB33/T 2540-2022) (Zhejiang Provincial Administration for Market Regulation, 2022). The assessment focused on eight key fields: organizational management, laboratory facilities and equipment, personnel management, management of pathogenic strains and biological samples, laboratory waste management, laboratory management and material labeling, security and confidentiality protocols, and other respects.

### 2.4 Post inspection remediation and closed-loop compliance verification

Following on-site inspections, identified non-conformities were documented in real time by assessment experts using a mobile application integrated with the Zhejiang Provincial Pathogenic Microorganism Laboratory Biosafety Assessment System. Non-compliant items were categorized based on the above standard, and digital assessment reports were generated after mutual confirmation. Inspected institutions were required to submit corrective action plans within 30 calendar days, with all corrective actions recorded in the centralized platform. The results of the corrective actions were subsequently verified by assessment panels, while the closed-loop verification of compliance was independently audited by the Lishui Municipal Health Commission to ensure procedural accountability.

### 2.5 Statistical analysis

Data analysis was performed using Microsoft Excel (Version 2503, Microsoft Corp) supplemented with the Data Analysis ToolPak. Raw datasets that did not contain non-compliant items were systematically cleaned; visual outputs (including bar charts, heat maps, horizontal bar charts, and bubble charts) were also generated to improve interpretability.

## 3 Results

### 3.1 Distribution of provincial quality control inspections for biosafety laboratories

As of 30 December 2024, a total of 261 registered biosafety laboratories (distributed across 140 institutions) were documented in Lishui through the Zhejiang Provincial Biosafety Online Supervision Systems. These included 6 BSL-1 laboratories, 253 BSL-2 laboratories, and 2 enhanced BSL-2 laboratories. According to the difference of institution types, the stratified random sampling was divided into 73 institutions: 23 in Liandu district; 12 in Qingtian county; 7 in Yunhe and Jingning counties; 6 in Longquan city; 5 in Qingyuan and Songyang counties; and 4 in Suichang and Yunhe counties (Table 1). According to the laboratories managed by different institutions, the stratified random sampling was divided into 128 laboratories: 38 in Liandu district; 24 in Qingtian county; 14 in Yunhe and Jingning counties; 13 in Longquan city; 9 in Qingyuan county; 8 in Songyang county; and 7 in Suichang county (Table 2).

**TABLE 1:** Distribution of quality control evaluation by laboratory-establishing institutions in Lishui (2023–2024).

Administrative districts	Healthcare delivery organizations	Public health authorities	Others	Total
Liandu district	17	2	4	23
Qingtian county	9	2	1	12
Jinyun county	6	1	0	7
Jingning county	5	2	0	7
Longquan city	4	1	1	6
Qingyuan county	4	1	0	5
Songyang county	3	2	0	5
Suichang county	2	2	0	4
Yunhe county	3	1	0	4
Total	53	14	6	73

**TABLE 2:** Distribution of quality control evaluation by individual laboratory in Lishui (2023–2024).

Administrative districts	Healthcare delivery organizations	Public health authorities	Others	Total
Liandu district	24	8	6	38
Qingtian county	20	3	1	24
Jinyun county	10	4	0	14
Jingning county	13	0	0	13
Longquan city	6	3	0	9
Qingyuan county	4	3	1	8
Songyang county	8	0	0	8
Suichang county	5	2	0	7
Yunhe county	3	4	0	7
Total	93	27	8	128

### 3.2 Analysis of biosafety laboratory assessment indicators: stratified by laboratory-establishing institutions and individual laboratory

**FIGURE 1:**
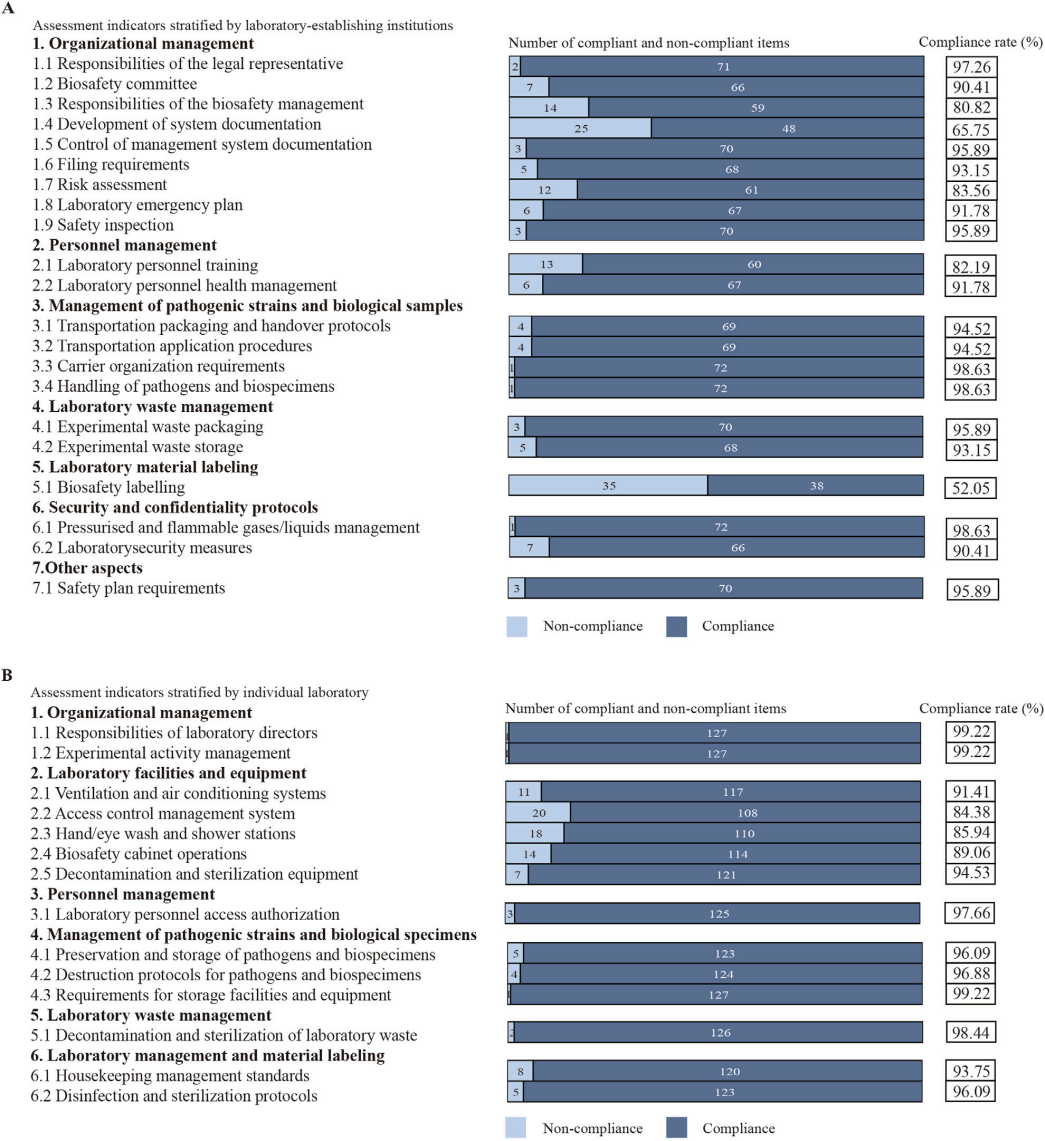
Analysis of assessment indicators. **(A)** Assessment indicators stratified by laboratory-establishing institutions. **(B)** Assessment indicators stratified by individual laboratory.

Based on assessment indicators stratified by laboratory-establishing institutions, 73 institutions exhibited low compliance rates (≤90%) in the following aspects: responsibilities of the biosafety management (80.82%; 59/73), development of system documentation (65.75%; 48/73), risk assessment (83.56%; 61/73), laboratory personnel training (82.19%; 60/73), and biosafety labelling (52.05%; 38/73) (Figure 1A). According to assessment indicators stratified by individual laboratory, 128 laboratories displayed low compliance rates (≤90%) in the following domains: access control management system (84.38%; 108/128), hand/eye wash and shower stations (85.94%; 110/128), and biosafety cabinet operations (89.06%; 114/128) (Figure 1B).

### 3.3 Analysis of assessment indicators: across administrative districts and in laboratories managed by these districts

**FIGURE 2:**
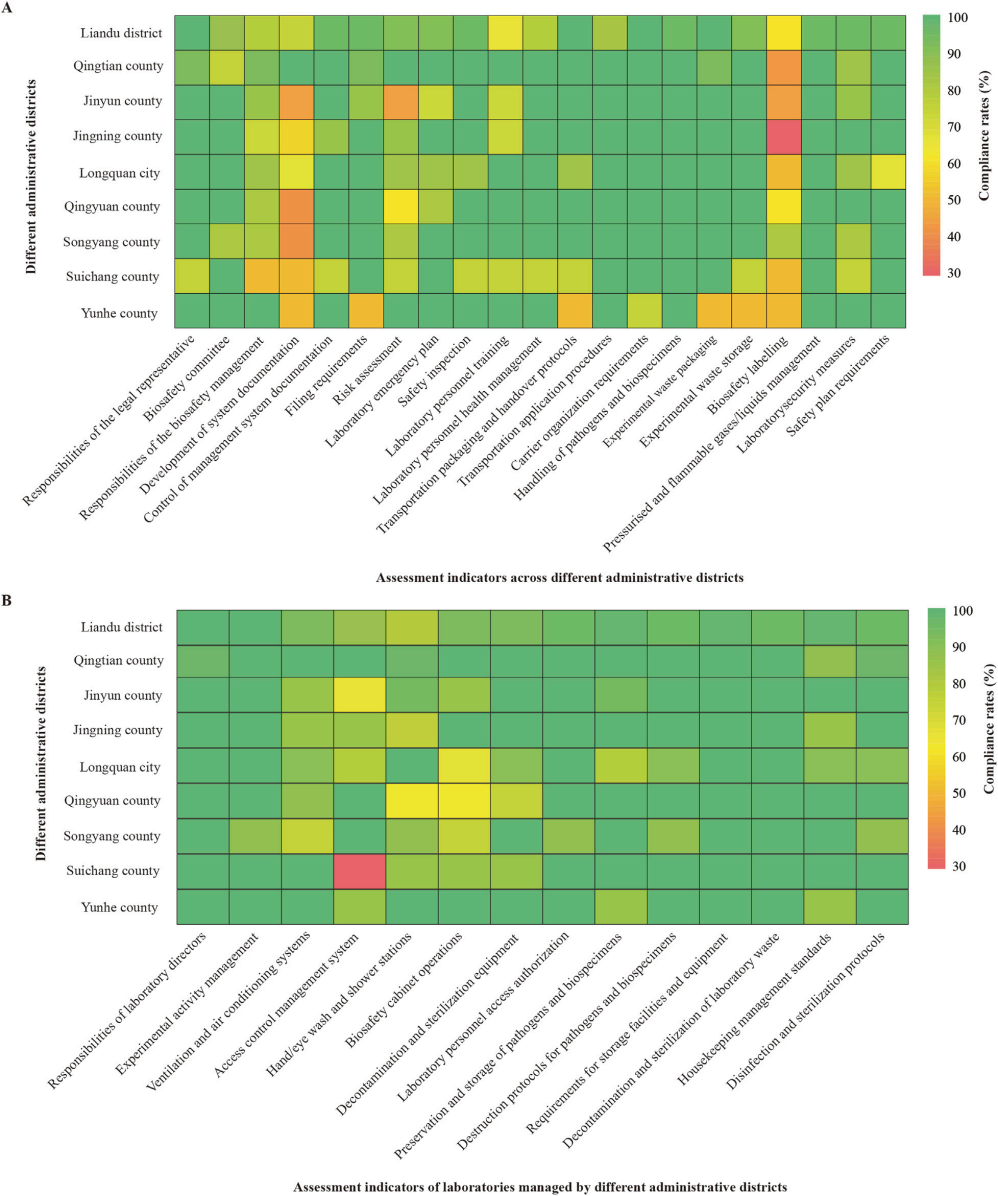
Analysis of compliance rates. **(A)** Compliance rates for assessment indicators across administrative districts (%). **(B)** Compliance rates for assessment indicators of laboratories managed by different administrative districts (%).

According to assessment indicators for cross-administrative districts, Liandu district showed low compliance rates (≤70%) in laboratory personnel training (65.22%) and biosafety labelling (60.87%); Qingtian county showed a low compliance rate in biosafety labelling (41.67%); Jinyun county showed low compliance rates in development of system documentation (42.86%), risk assessment (42.86%), and biosafety labelling (42.86%); Jingning county showed low compliance rates in development of system documentation (57.14%) and biosafety labelling (28.57%); Longquan city showed low compliance rates in development of system documentation (66.67%), biosafety labelling (50.00%), and safety plan requirements (66.67%); Qingyuan county showed low compliance rates in development of system documentation (40.00%), risk assessment (60.00%), and biosafety labelling (60.00%); Songyang county showed a low compliance rate in development of system documentation (40.00%); Suichang county showed low compliance rates in responsibilities of the biosafety management (50.00%), development of system documentation (50.00%), and biosafety labelling (50.00%); Yunhe county showed low compliance rates in development of system documentation (50.00%), filing requirements (50.00%), transportation packaging and handover protocols (50.00%), experimental waste packaging (50.00%), experimental waste storage (50.00%), and biosafety labelling (50.00%) (Figure 2A).

On the basis of assessment indicators for laboratories managed by these districts, Jinyun county demonstrated a low compliance rate (≤70%) in access control management system (64.29%); Longquan city demonstrated a low compliance rate in biosafety cabinet operations (66.67%); Qingyuan county demonstrated a compliance rate of 62.50% for both hand/eye wash stations and biosafety cabinet operations; Suichang county demonstrated a low compliance rate in access control management system (28.57%) (Figure 2B).

### 3.4 Analysis of assessment indicators: across different institutions and in laboratories managed by these institutions

**FIGURE 3:**
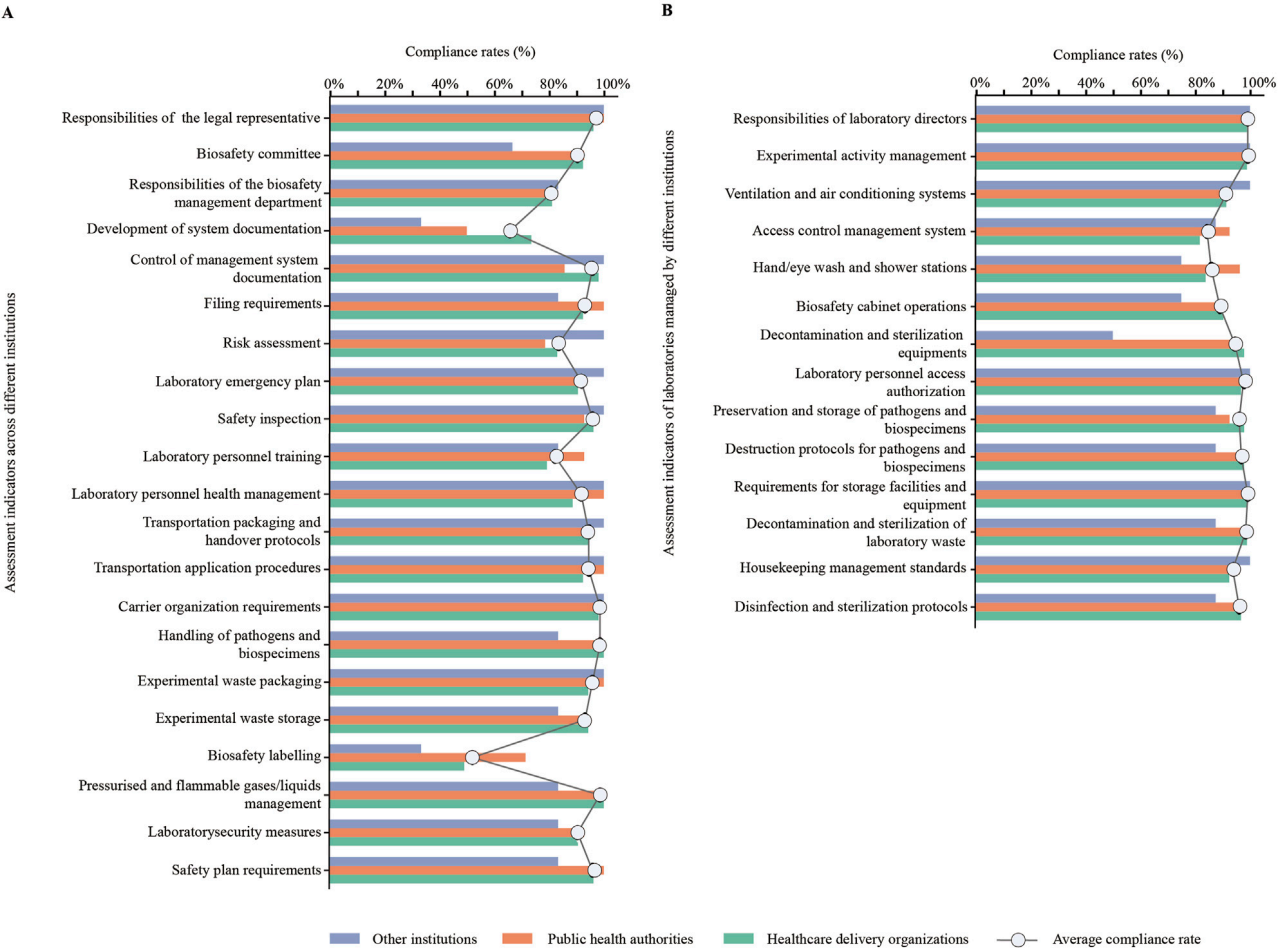
Analysis of compliance rates for assessment indicators. **(A)** Compliance rates for assessment indicators across different institutions (%). **(B)** Compliance rates for assessment indicators of laboratories managed by different institutions (%).

Through assessment indicators for cross-institutions, they presented low average compliance rates (≤90%) in responsibilities of the biosafety management department (80.82%), preparation of management system documentation (65.75%), risk assessment (83.56%), laboratory personnel training (82.19%), and biosafety labelling (52.05%). Additionally, HDOs presented a low compliance rate (≤70%) in biosafety labelling (49.06%); PHAs presented a low compliance rate in preparation of management system documentation (50.00%); other institutions presented low compliance rates in biosafety committee (66.67%), preparation of management system documentation (33.33%), and biosafety labelling (33.33%) (Figure 3A).

Based on assessment indicators for laboratories managed by these institutions, they exhibited low average compliance rates (≤90%) in access control management system (84.38%), hand/eye wash and shower stations (85.94%), and biosafety cabinet operations (89.06%). Moreover, other institutions presented a low compliance rate in decontamination and sterilization equipment (50.00%) (Figure 3B).

### 3.5 Analysis of non-compliance rates: across different institutions and for laboratories managed by these institutions in different administrative districts

**FIGURE 4:**
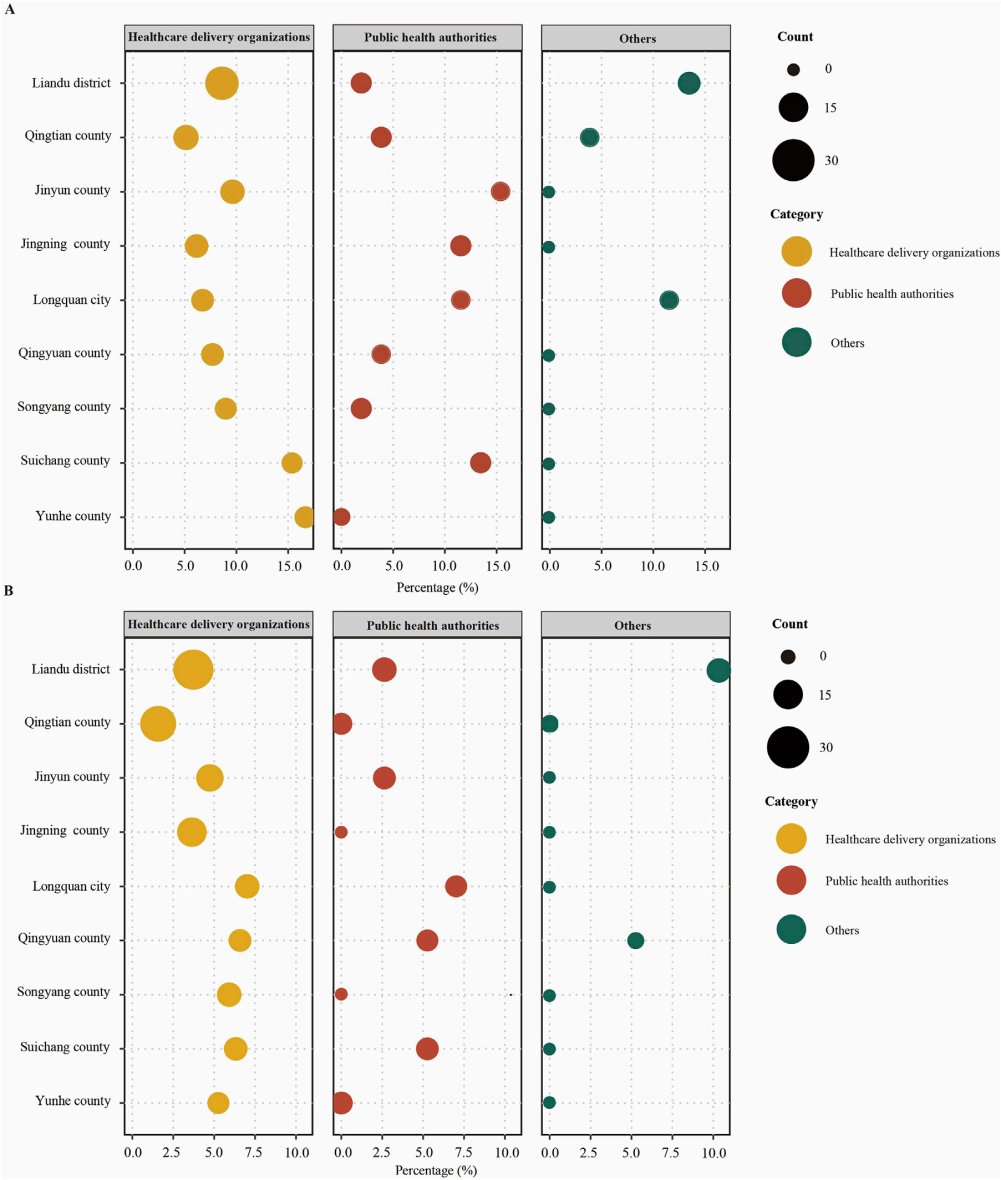
Analysis of non-compliance rates. **(A)** Non-compliance rates for assessment indicators of different institutions in different administrative districts (%). **(B)** Non-compliance rates for assessment indicators of laboratories managed by different institutions in different administrative districts (%).

The analysis of non-compliance rates across various institutions and administrative districts reveals high rates (≥10%) in specific areas. In Liandu district, the non-compliance rate is 13.46%, while in Longquan city, it is 11.54%. Furthermore, PHAs in Jinyun county show a rate of 15.38%, Jingning county has a rate of 11.54%, Longquan city again has 11.54%, and Suichang county has a rate of 13.46%. Additionally, HDOs in Suichang county and Yunhe county exhibit rates of 15.38% and 16.67%, respectively (Figure 4A). Based on non-compliance rates for laboratories managed by these institutions in different administrative districts, they demonstrated high non-compliance rates in other institutions in Liandu district (10.53%) (Figure 4B).

## 4 Discussion

This study presented the results of the biosafety quality control (QC) assessment in Lishui city from 2023 to 2024. In terms of the assessment indicators set up at the unit level, lower compliance rates were particularly evident in the development of system documentation (65.75%, 48/73) and biosafety labelling (52.05%, 38/73). This suggested that laboratory personnel lacked continuous training and failed to work in accordance with the latest national standards and codes. In addition, in low-resource biological laboratory environments, the availability of adequately trained and experienced laboratory personnel was limited, thereby reducing the level of laboratory biosafety management. Regarding the assessment indicators set up by individual laboratories, the combined pass rate for the 128 biosafety laboratories in Lishui city in terms of laboratory facilities and equipment (89.06%) was significantly lower than for the other assessment indicators. This reflected a lack of attention to this work by laboratory management, who failed to calibrate infrastructure and equipment in a timely manner, which increased the risk of damage. There also remained a risk that existing medical equipment might not comply with the latest biosafety standards (Siengsanan-Lamont et al., 2019).

Biosafety is one of the most important areas of concern in biomedical laboratories, and it has been reported that a risk-based approach to biosafety management can ensure the safety of laboratory personnel and the public by reducing the incidence of pathogen escapes (Kojima et al., 2018). A study revealed that 40% of instruments and equipment in hospitals in developing countries were inoperative due to a lack of timely calibration and maintenance (Trye et al., 2020). Unused medical equipment that contains dangerous and infectious pathogens poses a severe risk to both humans and the environment (Forti et al., 2020). Meanwhile, the existing medical equipment in some lower-middle-income or lower-income countries is far insufficient to meet their needs, and they continue to struggle to find adequate and appropriate instrumentation (Faust et al., 2020).

Notably, healthcare facilities in Yunhe county had significantly lower pass rates than other administrative districts for assessment indicators such as transportation packaging and handover protocols (50.00%), experimental waste packaging (50.00%), and storage (50.00%). This indicated that the specimen packaging system in Yunhe county urgently requires improvement. Biological specimens were vulnerable to external conditions, and factors like physical impact and prolonged transport time can significantly alter their structure and properties (Pillai and Raybould, 2023). Furthermore, inappropriate transport and storage conditions, such as extreme temperatures, may alter the inherent microbiota of specimens (Plebani, 2021).

In addition, PHAs (50.00%) and other institutions (33.33%) in Lishui city showed lower pass rates than HDOs (73.58%) for the assessment indicators in terms of system documentation. This revealed a lack of biosafety training for laboratory personnel. Several studies have emphasized the crucial role of biosafety training in improving understanding and application in laboratory settings (Back et al., 2022; Muneer et al., 2021). Other studies have similarly illustrated the significance of providing relevant training in the laboratory environment to raise awareness of biosafety and the importance of biosecurity measures (Butucel et al., 2022). Besides basic theoretical courses, practical sessions on specific themes can significantly enhance laboratory personnel’s learning in biosafety (Cruz et al., 2015).

To address these challenges, it was essential to enhance individual awareness of biosafety by quantifying the performance indicators of laboratory personnel in this area (Blatter et al., 2023). Additionally, biosafety could be promoted by identifying deficiencies in the existing biosafety system when resources are limited (Xue and Shang, 2022). The efficiency of departmental cooperation should be improved by introducing smart technologies to achieve seamless management and form action teams capable of rapid response (Peretti et al., 2022). While not all medical institutions in Lishui city were included, the study encompassed various institutions across the city’s nine counties and districts. This approach provides a more accurate representation of the overall laboratory capacity.

## 5 Conclusion

This study revealed deficiencies in the pathogenic microbiological laboratories in Lishui through analysis of data from the Zhejiang Provincial Online Laboratory Biosafety Supervision System. Future efforts should focus on strengthening laboratory personnel training and implementing biosafety management responsibilities to ensure safe and regulation-compliant operations in laboratories handling pathogenic microorganisms. Additionally, it is essential to enhance interdisciplinary cooperation and innovative strategies should be continuously explored to address the evolving biosafety challenges.

## Data Availability

The raw data supporting the conclusions of this article will be made available by the authors, without undue reservation.

## References

[B1] Alderman T. S. Carpenter C. B. McGirr R. (2018). Animal research biosafety. Appl. Biosaf. 23, 130–142. 10.1177/1535676018776971

[B2] Back J. B. Martinez L. Nettenstrom L. Sheerar D. Thornton S. (2022). Establishing a biosafety plan for a flow cytometry shared resource laboratory. Cytom. A 101, 380–386. 10.1002/cyto.a.24524 PMC9081124 35037390

[B3] Blacksell S. D. Dhawan S. Kusumoto M. Le K. K. Summermatter K. O’Keefe J. (2023). The biosafety research road map: the search for evidence to support practices in human and veterinary laboratories. Appl. Biosaf. 28, 64–71. 10.1089/apb.2022.0040 37342514 PMC10277988

[B4] Blatter T. Nagabhushana P. Schär D. Ackermann J. Cadamuro J. Leichtle A. (2023). Statistical learning and big data applications. J. Lab. Med. 47, 181–186. 10.1515/labmed-2023-0037

[B5] Brown C. S. Zwetyenga J. Berdieva M. Volkova T. Cojocaru R. Costic N. (2015). New policy-formulation methodology paves the way for sustainable laboratory systems in Europe. Public Health Panor. 1, 41–47. 10.5040/9781474216142.ch-003

[B6] Butucel E. Balta I. McCleery D. Morariu F. Pet I. Popescu C. A. (2022). Farm biosecurity measures and interventions with an impact on bacterial biofilms. Agriculture 12, 1251. 10.3390/agriculture12081251

[B7] Cao C. (2021). China’s evolving biosafety/biosecurity legislations. J. Law Biosci. 8, lsab020. 10.1093/jlb/lsab020 34221436 PMC8245076

[B8] Chen F. Zhang Z. Ding C. Wu X. (2020). Analysis of global biosafety strategy and recommendations to China. Bull. Chin. Acad. Sci. 35, 135–146. Available online at: https://bulletinofcas.researchcommons.org/journal/vol35/iss2/10/.

[B9] Cruz C. P. Cruz J. P. Bakr S. A. A. Thazha S. K. (2015). Biosafety knowledge and perceptions of clinical laboratory science educators in Shaqra University. J Infect Public Heal. 8, 398. 10.1016/j.jiph.2015.04.008

[B10] Davies J. Abimiku A. Alobo M. Mullan Z. Nugent R. Schneidman M. (2017). Sustainable clinical laboratory capacity for health in Africa. Lancet Glob. Health 5, e248–e249. 10.1016/S2214-109X(17)30024-4 28108137

[B11] Edwards S. Jeggo M. H. (2010). Governance and management of veterinary laboratories. Rev. Sci. Tech. Off. Int. Epiz. 31, 493–503. 10.20506/rst.31.2.2140 23413729

[B12] Faust L. Zimmer A. J. Kohli M. Saha S. Boffa J. Bayot M. L. (2020). SARS-CoV-2 testing in low- and middle-income countries: availability and affordability in the private health sector. Microbes Infect. 22, 511–514. 10.1016/j.micinf.2020.10.005 33065265 PMC7553871

[B13] Forti V. Baldé C. Kuehr R. Bel G. (2020). The global E-waste monitor 2020. Available online at: https://ewastemonitor.info/wp-content/uploads/2020/11/GEM_2020_def_july1_low.pdf (Accessed March 7, 2025).

[B14] Gao Y. C. Hu X. L. Chen W. Gu H. (2024). Practice and prospect of intelligent supervision of biosafety laboratories in Zhejiang Province. Exp. Technol. Manag. 41, 232–238. 10.16791/j.cnki.sjg.2024.01.032

[B15] Global Health Security Agenda (2014). Action packages, global health security agenda (GHSA). Available online at: https://globalhealthsecurityagenda.org/action-packages/ (Accessed July 19, 2021).

[B16] Hamilton K. Lasley J. Harper D. (2018). Improving sustainability to avoid laboratory disasters. Responding to concerns about the sustainability of laboratories, the OIE, together with Chatham House, is working to explore solutions. Bull. WOAH. Available online at: https://bulletin.woah.org/?p=3021 (Accessed March 7, 2025).

[B18] Klotz L. C. Sylvester E. J. (2014). The consequences of a lab escape of a potential pandemic pathogen. Front. Public Health 2, 116. 10.3389/fpubh.2014.00116 25157347 PMC4128296

[B19] Kojima K. Booth C. Summermatter K. Bennett A. Heisz M. Blacksell S. (2018). Riskbased reboot for global lab biosafety. Science 360, 260–262. 10.1126/science.aar2231 29674576

[B20] Lentzos F. Koblentz G. D. (2023). High consequence bio labs: growing risks and lagging governance. Release Glob. BioLabs 2023 Rep. Available online at: https://static1.squarespace.com/static/62fa334a3a6fe8320f5dcf7e/t/6414862df5bf8f14cec76598/1679066680422/Global-Biolabs-2023-Report.pdf (Accessed March 7, 2025).

[B22] Lippi G. Plebani M. (2020). The critical role of laboratory medicine during coronavirus disease 2019 (COVID-19) and other viral outbreaks. Clin. Chem. Lab. Med. 58, 1063–1069. 10.1515/cclm-2020-0240 32191623

[B23] Min Y. Perucci F. (2020). UN/DESA policy brief #81: impact of COVID-19 on SDG progress: a statistical perspective. Available online at: https://desapublications.un.org/policy-briefs/undesa-policy-brief-81-impact-covid-19-sdg-progress-statistical-perspective (Accessed March 7, 2025).

[B24] Mohsen D. M. Dpagh A. N. Dpagh A. (2015). Containment evidence-based biosafety: effectiveness of microbiological measures for the handling of mycobacterium isolates in the laboratories. Egypt J. Chem. Environ. Health 1, 1049–1057. 10.21608/ejceh.2015.254005

[B25] Morrison J. S. Simoneau M. (2023). Eight commonsense actions on biosafety and biosecurity. CSIS Briefs. Available online at: www.CSIS.org (Accessed March 7, 2025).

[B26] Muneer S. Kayani H. A. Ali K. Asif E. Zohra R. R. Kabir F. (2021). Laboratory biosafety and biosecurity related education in Pakistan: engaging students through the Socratic method of learning. J. Biosaf. Biosecur 3, 22–27. 10.1016/j.jobb.2021.03.003

[B27] Pašalić A. Šegalo S. Maestro D. Biščević-Tokić J. Jogunčić A. Panjeta M. (2022). Risk assessment in biomedical laboratories-occupational safety and health aspects. J. Health Sci. 12, 231–237. 10.17532/jhsci.2022.2044

[B28] Peretti O. Spyridis Y. Sesis A. Efstathopoulos G. Lytos A. Lagkas T. (2022). “Augmented reality training, command and control framework for first responders,” in 2022 7th south-east Europe design automation, computer engineering, computer networks and social media conference (SEEDA-CECNSM). IEEE, 1–5. Available online at: https://ieeexplore.ieee.org/document/9932966 (Accessed March 7, 2025).

[B29] Pillai S. P. Raybould A. (2023). Editorial: insights in biosafety and biosecurity 2022: noveldevelopments, current challenges, and future perspectives. Front. Bioeng. Biotechnol. 10, 1118506. 10.3389/fbioe.2022.1118506 36714616 PMC9878383

[B30] Plebani M. (2021). Drone transport of biological samples: an open issue. Clin. Chem. LabMed 59, 1745–1746. 10.1515/cclm-2021-0811 34318652

[B31] Qasmi S. A. Standley C. Mohsin S. Sarwar S. Malik L. Aziz F. (2022). Effectiveness of international virtual training on biorisk management in the context of COVID-19. Front. Public Health 10, 888097. 10.3389/fpubh.2022.888097 36339241 PMC9627603

[B32] Siengsanan-Lamont J. Blacksell S. D. (2018). A review of laboratory-acquired infections in the Asia-Pacific: understanding risk and the need for improved biosafety for veterinary and zoonotic diseases. Trop. Med. Infect. Dis. 3, 36. 10.3390/tropicalmed3020036 30274433 PMC6073996

[B33] Siengsanan-Lamont J. Kamolsiripichaiporn S. Ruanchaimun S. Patchimasiri T. Jongrakwattana B. Blacksell S. D. (2019). Biosafety and biosecurity challenges facing veterinary diagnostic laboratories in lower-middle income countries in southeast Asia: a case study of Thailand. Appl. Biosaf. 24, 220–230. 10.1177/1535676019869771 32655327 PMC7323819

[B34] Trye A. Maloney M. Jalal E. Parikh R. Jalloh S. Johnston P. F. (2020). A post-Donation survey to assess the appropriateness of medical supply donations to freetown, Sierra Leone following the ebola crisis. Cureus 12, e7228. 10.7759/cureus.7228 32280571 PMC7145378

[B35] World Health Organization (2020). “Laboratory biosafety manual,” in Laboratory design and maintenance. 4th edition. Available online at: https://www.who.int/publications/i/item/9789240011397 (Accessed March 7, 2025).

[B36] World Health Organization (2020). Laboratory biosafety manual 4th edition. Available online at: https://www.who.int/publications/i/item/9789240011311 (Accessed March 7, 2025). 24404640

[B37] Xue Y. Shang L. (2022). Towards better governance on biosafety and biosecurity: china’s advances and perspectives in medical biotechnology legislation. Front. Bioeng. Biotechnol. 10, 939295. 10.3389/fbioe.2022.939295 35860327 PMC9289183

[B38] Zhejiang Provincial Administration for Market Regulation (2022). Specification for the evaluation of biosafety laboratory management. (DB33/T2540-2022). Available online at: https://zjjcmspublic.oss-cn-hangzhou-zwynet-d01-a.internet.cloud.zj.gov.cn/jcms_files/jcms1/web3397/site/attach/-1/2210101724274731707.pdf (Accessed March 7, 2025).

